# Sleep Health of Athletes of Different Sports: Translation and Validation of the Athlete Sleep Screening Questionnaire to Portuguese

**DOI:** 10.1111/jsr.70349

**Published:** 2026-05-11

**Authors:** João Barreira, Júlio A. Costa, João Brito, Vasco Cardoso, Cátia Reis

**Affiliations:** ^1^ Research Center in Sports Sciences, Health Sciences and Human Development, CIDESD University of Maia, ISMAI Maia Portugal; ^2^ FPF Academy Portuguese Football Federation Oeiras Portugal; ^3^ CIPER, Faculdade de Motricidade Humana Universidade de Lisboa Lisboa Portugal; ^4^ CRC‐W Faculdade de Ciências Humanas Universidade Católica Portuguesa Lisbon Portugal; ^5^ ISAMB Faculdade de Medicina Universidade de Lisboa Lisbon Portugal

**Keywords:** chronotype, health, monitoring, sports, well‐being

## Abstract

This study aimed to translate and validate the Athlete Sleep Screening Questionnaire (ASSQ) into Portuguese and assess the sleep health of athletes using the ASSQ. Translation followed established scientific guidelines. A sample of 246 Portuguese athletes from various sports completed the ASSQ, the Pittsburgh Sleep Quality Index (PSQI) and the Reduced Morningness–Eveningness Questionnaire (r‐MEQ). Exploratory and confirmatory factor analysis supported two factors for the Sleep Difficulty Score (SDS) and chronotype subscales. Model fit indices indicated excellent fit for SDS and chronotype. Internal consistency was acceptable for SDS (*α* = 0.58) and chronotype (*α* = 0.54). Mean SDS was 5.7, and 22% of the sample were found to have sleep problems. Specifically, 16% were classified with moderate sleep problems and 6% with severe sleep problems. No significant differences in SDS categories were found across sex, sports type, sports environment, competitive level or age group. Travel‐related sleep disturbances were reported by 20% of athletes, and 11% experienced performance issues after travelling. Chronotype differed by sports type, sports environment, competitive level and age group, but not by SDS categories. Additionally, morning chronotypes reported more travel‐related sleep disturbances (*p* = 0.045). The Portuguese ASSQ showed strong validity and acceptable reliability. Cohort analysis revealed that a substantial proportion of Portuguese athletes reported having sleep problems and some would benefit from specific interventions intended to improve sleep.

## 1 Introduction

Adequate sleep is essential for optimal athletic performance, recovery and general well‐being. Emerging evidence highlights that not only sleep duration and quality, but also dimensions such as timing, efficiency and regularity are vital to sleep health (Consensus Conference Panel 2015; Walsh et al. 2020). While a consensus remains to be established on the recommended amount of sleep an athlete should obtain (Walsh et al. 2020), healthy adults are recommended to sleep from 7 to 9 h per night, while for younger populations (14–17 years old) 8–10 h is recommended (Consensus Conference Panel 2015). However, recent evidence points to adult athletes sleeping, on average, 6.7 h even though their perceived sleep duration need was found to be, on average, 8.3 h (Sargent et al. 2021).

The sports science community and, recently, the International Olympic Committee recognise the importance of sleep and that sleep deficiency and sleep disturbances seriously affect athletes' health and physical performance (Bergeron et al. 2015; Gouttebarge et al. 2021; Halson 2014). Therefore, implementing routine assessment, identification and personalised interventions for athletes' sleep health is important in any sports context (Bender et al. 2018). As such, having valid and reliable tools to assess sleep difficulties in this special population is fundamental (Samuels et al. 2016). While polysomnography is considered the gold standard in sleep monitoring (Driller et al. 2023), non‐evasive alternatives such as actigraphy and sleep‐specific questionnaires can also provide important information about sleep habits and problems (Halson 2019). Clinical interviews with sleep specialists, supported by objective sleep assessment methods such as polysomnography or actigraphy, can provide high‐quality diagnostic information. However, they can be costly and often not easily available. In contrast, questionnaires are a valuable subjective tool that can provide valuable information upon sleep behaviours in large groups.

The Pittsburgh Sleep Quality Index (PSQI) (Buysse et al. 1989) is the primary questionnaire used to assess sleep quality in athletes; however, to our best knowledge, it has not yet been validated in athlete‐specific populations (Bender et al. 2018; Samuels et al. 2016; Bender and Samuels 2017). Furthermore, it lacks information specific to athletes and sports practice and has shown poor agreement rates with clinical assessment of a sleep medicine physician (Samuels et al. 2016). Another tool which is progressively more commonly used is the Athlete Sleep Behaviour Questionnaire (ASBQ), used to identify maladaptive sleep behaviours in athletes (Driller et al. 2018). The ASBQ shows promise in differentiating sleep behaviours between athletes and controls (i.e., non‐athletes) but is still in development to determine valid cut‐off points that are not based on the author's speculations (Bender et al. 2018). Additionally, the ASBQ is not intended to be used as a clinical sleep screening tool but instead to inform sleep hygiene recommendations specifically for athletes.

Quite recently, the Athlete Sleep Screening Questionnaire (ASSQ) was developed (Samuels et al. 2016). It intends to function as a sleep screening tool to detect clinically significant sleep disturbances and daytime dysfunction and to provide interventions based on the type and severity of the problem that is detected in an athlete population (Samuels et al. 2016). In summary, it is a 15‐item questionnaire that assesses sleep and circadian factors of sleep duration, sleep quality, symptoms of insomnia and chronotype with a timeframe of ‘over the recent past’ (Samuels et al. 2016). The sum of the first seven items comprises the sleep difficulty score (SDS), which is categorised into four intensities of the symptoms: none, mild, moderate and severe. One dimension of the questionnaire (four questions) aims to classify individual's chronotype. The remaining questions are intended to assess other important factors of sleep‐disordered breathing and sleep and performance issues during travelling. Based on previous studies, 13% of 349 studied athletes were classified as having moderate to severe clinical sleep problems and required further interventions (Samuels et al. 2016).

In addition to its practical usefulness, the ASSQ has been used to characterise sleep and assess sleep disturbances in different athletic populations (Bender et al. 2018; Rabin et al. 2020; Zhang et al. 2022). Studies have shown that different ethnic groups of athletes have different characteristics (Huskey et al. 2021). For Portuguese athletes, to date, there's no study describing the sleep and possible sleep disturbances for this specific group. As such, it is of interest to not only translate and validate the ASSQ in the Portuguese language but also to characterise and assess sleep disturbances of Portuguese athletes through the Portuguese version of the ASSQ. Considering past research, we hypothesised that a great proportion of Portuguese athletes would present, at least, mild sleep disturbances and sleep duration below current recommendations for the specific age groups.

## 2 Methods and Materials

### 2.1 Design

The aim of this study was to translate and validate the Athlete Sleep Screening Questionnaire to European Portuguese and to assess sleep difficulties and disturbances in athletes in Portugal. First, the ASSQ was translated to Portuguese according to recommended methodologies presented in Supporting Information S1. Then, athletes from various sports and competitive levels were invited to complete a series of questionnaires designed to assess the validity of the Portuguese version of the ASSQ. This study was approved by the Ethics Committee of the Portugal Football School (CEPFS 19.2023) as a low‐risk study and was conducted in accordance with the ethical standards outlined in the Declaration of Helsinki. No sensitive data were collected; participation was anonymous and voluntary, and all participants were Portuguese speakers living in Portugal and provided informed consent before taking part in the study.

### 2.2 Participants

Portuguese athletes were recruited to complete a battery of questionnaires aimed at assessing the psychometric properties of the Portuguese version of the ASSQ. Invitations were sent by email, through national federations' networks, clubs and recruitment also took place onsite during official competitions and sports events. All questionnaires were answered via survey (Microsoft Forms, Microsoft). To be included in the formal data analysis, the following inclusion criteria were considered: (1) athletes had to be aged 14 years or older at the time of completing the questionnaires and (2) athletes had to be classified as at least tier 2—trained/developmental according to the framework proposed by McKay et al. (2022). The lower age threshold was defined because the ASSQ includes items related to travel for training and competition, which become more relevant as athletes begin to participate in higher‐level competitive contexts. In addition, because data collection was conducted anonymously through an online link, the approved ethical procedures required electronic informed consent to be provided directly by the participant before accessing the questionnaire. Under these conditions, younger children were not included.

Initially, a total of 276 athletes completed the questionnaire (mean ± SD age: 24.8 ± 11.7). The characteristics of the final sample are presented in the ‘Results’ section.

#### 2.2.1 Athlete Sleep Screening Questionnaire

The ASSQ is a 16‐item questionnaire that was developed with the aim of examining six key sleep parameters: sleep quality, sleep duration, circadian preference, insomnia, sleep disturbances while travelling and sleep‐disordered breathing (i.e., obstructive sleep apnoea) (Samuels et al. 2016). A sleep difficulty score (SDS) was also developed within the questionnaire, based on the response to items 1, 3, 4, 5 and 6 with a higher score indicating higher sleep difficulty (and poorer sleep). Subject's chronotype is also calculated, as a subscale of the ASSQ, based on the responses to item 7, 8, 9 and 10.

The original ASSQ study and past studies which applied the ASSQ have classified chronotype as follows: 0–4 evening type and above 5 would be the remaining (morning and intermediate types). Considering that it is also relevant to specifically identify evening, intermediate and morning types, we have employed quartile and percentile analysis to define subjects' chronotype:

- *Quartile analysis:* a score from 0 to 6 (minimum to 25th) was considered evening type; a score from 7 to 9 (25th to 75th) was considered intermediate type; a score from 9 to 14 (75th to maximum value) was considered morning type.
- *Percentile analysis:* a score from 0 to 4 (minimum to 10th) was considered extreme evening type; a score from 10 to 14 (90^th^ to maximum value) was considered extreme morning type.

As such, the ASSQ does not result in a total score and instead consists of two subscales: SDS and chronotype. Lastly, there are six items that are not included in the scoring process. These items (11–14) act as modifiers, providing specific education and recommendations for athletes. Items 2 (napping), 15 (caffeine use) and 16 (use of electronic devices before bed) aim to inform athletes about strategies and potential sleep disruption behaviours. A detailed explanation of the ASSQ is provided in Supporting Information S2.

#### 2.2.2 Additional Questionnaires

*Pittsburgh Sleep Quality Index (PSQI)*: The PSQI is a 19‐item questionnaire designed to measure sleep quality and disturbances over the previous four weeks (Buysse et al. 1989). The Portuguese version of the PSQI in the current study is the one used in (Del Rio et al. 2017).

Morningness–Eveningness reduced version (r‐MEQ): circadian typology was also assessed via the Portuguese version of the reduced version of the Horne and Ostberg Morningness–Eveningness Questionnaire (Loureiro and Garcia‐Marques 2015). Scores on the r‐MEQ range from 4 to 25, and subjects are classified as morning‐type (scores from 18 to 25), evening‐type (scores from 12 to 17) or neither type (scores from 4 to 11).

### 2.3 Statistical Analyses

All statistical analyses were conducted using R (version 2023.12.1 + 402). The significance level was set at *p* < 0.05. Descriptive statistics (mean, standard deviation, skewness and kurtosis) were computed for all items to assess normality. Internal consistency of the ASSQ subscales was assessed using Cronbach's *α* and McDonald's Ω. Exploratory factorial analysis (EFA) and confirmatory factorial analysis (CFA) were performed. A detailed statistical analysis description is presented in Supporting Information S3, together with the respective results.

#### 2.3.1 Assessment of Sleep Disturbances in Portuguese Athletes

The Shapiro–Wilk test was used to check the normality of data distribution. If data were not normally distributed, then non‐parametric analysis methods were used. Descriptive statistics were used to present continuous data as the mean and 95% confidence interval, and categorical data as frequencies (*n*) and proportions (%). Mann–Whitney test was used to compare SDS between sex, sports type (team vs. individual sport), and sports environment group (indoor vs. outdoor sport). Kruskal–Wallis tests were used to compare the SDS between competitive level and age groups. A cross‐table Chi‐squared (*χ* ^2^) or Fisher's exact test was used where appropriate to assess the SDS category differences of sex, different sports type, sports environment, sports level and chronotype, and Cramer's V for estimation of effect size (small, medium or large).

SDS and ASSQ chronotype were compared between different groups, including sex, competitive level, type of sport and sport environment. Considering the distributions, group differences were assessed using non‐parametric tests (Kruskal–Wallis). Effect sizes were reported to quantify the magnitude of differences. Spearman's Rank Correlations were calculated between the total questionnaire score and other validated instruments measuring similar constructs. Correlations above 0.50 were considered strong, while values between 0.30 and 0.50 were interpreted as moderate (Rosnow et al. 2000).

## 3 Results

### 3.1 Sample Characteristics

From the initial 276 answers, 242 answers were retained for analysis. Answers were excluded based on the competitive tier classification (*n*, tier 0 = 5, tier 1 = 13) and age (*n*, < 14 years old = 21, > 65 years old = 2).

Table 1 presents the characteristics of the participants in the study. Of the final included Portuguese participants (*n* = 242), 103 were females (37%). The mean age of the participants was 25.5 ± 12.0 years and ranged from 14 to 59 years. A total of 22 different sports were included in the study, and 82 participants practiced an individual sport and 160 were members of a team sport. According to the athlete classification framework (McKay et al. 2022), participants were classified as tier 2—trained/developed (*n* = 71), tier 3—highly trained/national level (*n* = 152), tier 4—elite/international level (*n* = 16) and tier 5—world class (*n* = 3).

**TABLE 1:** Sample characteristics.

Sport	*n*	Female (*n*, %)	Age (mean ± SD)
Soccer	57	14 (25)	20.2 ± 3.6
Volleyball	37	35 (95)	18.9 ± 4.1
Triathlon	32	7 (20.6)	45.0 ± 7.1
Canoeing	24	8 (30.0)	25.2 ± 13.7
Futsal	20	1 (5)	17.2 ± 3.3
Basketball	21	9 (43)	20.6 ± 5.4
Handball	10	5 (50.0)	19.1 ± 3.3
Swimming	5	2 (40.0)	17.0 ± 1.2
Rugby	5	3 (60.0)	27.5 ± 2.8
Sailing	5	0 (0.0)	21.2 ± 4.7
Athletics	4	0 (0.0)	21.0 ± 3.2
Cycling	3	1 (33)	33.7 ± 19.9
Dance	3	2 (67)	16.0 ± 1.0
Combat Sports	3	1 (33)	25.0 ± 1.0
Padel	3	1 (33)	30.0 ± 6.6
Speed Skating	3	2 (67)	15.7 ± 1.2
CrossFit	2	1 (50.0)	31.0 ± 11.3
Badminton	1	0 (0.0)	15
American Football	1	0 (0.0)	29
Motorcross	1	0 (0.0)	20
Parasurf	1	0 (0.0)	44
Table Tennis	1	0 (0.0)	28
Total	242	92 (38)	24.1 ± 10.9

### 3.2 Questionnaire Validity, Reliability and Internal Consistency

The validity of the SDS and Chronotype subscales was confirmed through EFA and subsequent CFA analysis. SDS subscale: *χ* ^2^(2) = 2.97, *p* = 0.563, DF = 6, CFI = 1.0, TLI = 1.057, RMSEA = 0.0 (90% CI 0.0, 0.169), SRMR = 0.017. Chronotype subscale: *χ* ^2^(1) = 3.331, *p* = 0.068, DF = 6, CFI = 0.98, TLI = 0.883, RMSEA = 0.0 (90% CI 0.0, 0.250), SRMR = 0.027.

Reliability analysis results for each questionnaire and sub‐scales are presented in Table 2. Results indicated that the internal consistency of SDS (Cronbach's alpha = 0.66 [95% CI 0.49, 0.67]) and chronotype (Cronbach's alpha = 0.66 [0.58, 0.74]) were below the defined suitable criterion (> 0.7). For further detail of the translation and validation process, and questionnaire reliability, see Supporting Information S4.

**TABLE 2:** Reliability and convergent validity of the ASSQ SDS, ASSQ chronotype subscale, PSQI and r‐MEQ.

Questionnaire	Number of items	Cronbach's alpha (95% CI)	Ω	AVE	CR
ASSQ (SDS)	5	0.58 (0.48, 0.65)	0.58	0.23	0.57
ASSQ (Chronotype)	4	0.54 (0.43, 0.61)	0.60	0.33	0.59
PSQI	7^a^	0.62 (0.56, 0.69)	0.65	0.22	0.65
r‐MEQ	5	0.64 (0.57, 0.70)	0.67	0.30	0.68

Abbreviations: Ω, McDonald omega coefficient; ASSQ, athlete sleep screening questionnaire; AVE, average variance extracted; CI, confidence intervals; CR, construct reliability; PSQI, Pittsburgh sleep quality index; SDS, sleep difficulty score.

^a^ PSQI reliability was calculated based on the seven components of the questionnaire.

Analysing the PSQI seven components indicated (Cronbach's alpha = 0.63 [95% CI 0.49, 0.67]) and r‐MEQ (Cronbach's alpha = 0.66 [0.58, 0.74]) were below the defined suitable criterion (> 0.7). A strong, positive correlation was found between SDS and PSQI scores (*r* = 0.61 [95% CI 0.51, 0.67]). A strong, positive correlation was found between SDS chronotype subscale and the r‐MEQ scores (*r* = 0.85 [0.81, 0.88]).

### 3.3 Sample Sleep Disturbances and Difficulties Assessment With the ASSQ

#### 3.3.1 Sleep Difficulty Score and Sleep Problem Category

Participants' SDS mean score was 5.7, with no significant differences between male and female athletes. There were significant differences in SDS between adolescents and young adults (*p* < 0.001) (Table 3). No differences were found in SDS across sports type, sports environment and competitive level (Table 3). A Chi‐square test of independence (or Fisher's exact test when assumptions were not met) revealed several significant associations. Sports type was significantly related to trouble staying asleep (*χ* ^2^ = 11.05, *p* = 0.011) and quartile chronotype (*χ* ^2^ = 30.54, *p* < 0.001). Sports environment was also associated with quartile chronotype (*χ* ^2^ = 20.57, *p* < 0.001), and competitive level with quartile chronotype (*χ* ^2^ = 15.31, *p* = 0.017). Finally, age group was significantly related to sleep duration (*χ* ^2^ = 43.34, *p* < 0.001), trouble staying asleep (*χ* ^2^ = 24.99, *p* < 0.01) and quartile chronotype (*χ* ^2^ = 27.16, *p* < 0.001) (Table 2).

**TABLE 3:** Participants demographics and sleep characteristics.

	Total	Sex		Sports type		Sports environment		Competitive level				Age group		
		Male	Female	Team sport	Individual sport	Indoor sport	Outdoor sport	Tier 2	Tier 3	Tier 4	Tier 5	Adolescent	Young adult	Adult
	Mean (95% CI)	Mean (95% CI)	Mean (95% CI)	Mean (95% CI)	Mean (95% CI)	Mean (95% CI)	Mean (95% CI)	Mean (95% CI)	Mean (95% CI)	Mean (95% CI)	Mean (95% CI)	Mean (95% CI)	Mean (95% CI)	Mean (95% CI)
Age	24.1 (22.7, 25.5)	25.3 (23.2, 27.3)	22.0 (20.3, 23.8)	19.8 (19.1, 20.5)	32.5 (29.3, 35.6)	19.2 (18.3 20.1)	27.7 (25.6, 29.8)	26.5 (23.5, 29.6)	23.4 (21.8, 25.1)	18.7 (16.8, 20.6)	26.7 (18.7, 34.7)	15.8 (15.6, 16.0)	20.9 (20.4, 21.4)	38.9 (36.4, 41.4)
SDS	5.7 (5.4, 6.0)	5.7 (5.3, 6.1)	5.7 (5.2, 6.3)	5.5 (5.2, 5.9)	6.0 (5.4, 6.7)	5.7 (5.2, 6.2)	5.7 (5.3, 6.1)	6.1 (5.5, 6.8)	5.5 (5.2, 5.9)	5.1 (3.8, 6.3)	6.7 (0.4, 12.9)	**4.9 (4.6, 5.4)**	**5.5 (4.9, 6.0)**	6.9 (6.1, 7.6)

	*n* (%)	*n* (%)	*n* (%)	*n* (%)	*n* (%)	*n* (%)	*n* (%)	*n* (%)	*n* (%)	*n* (%)	*n* (%)	*n* (%)	*n* (%)	*n* (%)
Nocturnal sleep duration														
5–6 h	76 (31)	42 (28)	34 (37)	43 (27)	33 (40)	32 (31)	44 (32)	20 (28)	52 (34)	3 (19)	1 (33)	20 (24)	22 (23)	34 (52)
6–7 h	98 (41)	58 (39)	40 (43)	69 (43)	29 (35)	41 (40)	57 (41)	31 (44)	57 (38)	10 (62)	0 (0)	**46 (56)**	**38 (40)**	**14 (21)**
7–8 h	39 (16)	28 (19)	11 (12)	30 (19)	9 (11)	17 (17)	22 (16)	9 (13)	25 (16)	3 (19)	2 (67)	**13 (16)**	**22 (23)**	**4 (6)**
8–9 h	26 (11)	20 (13)	6 (7)	16 (10)	10 (12)	12 (12)	14 (10)	10 (14)	16 (11)	0 (0)	0 (0)	**2 (2)**	**10 (11)**	**14 (21)**
> 9 h	3 (1)	2 (1)	1 (1)	2 (1)	1 (1)	1 (1)	2 (1)	1 (1)	2 (1)	0 (0)	0 (0)	1 (1)	2 (2)	0 (0)
Sleep satisfaction														
Very satisfied	117 (48)	72 (48)	45 (49)	72 (45)	45 (55)	44 (43)	73 (53)	31 (44)	76 (50)	9 (56)	1 (33)	43 (52)	40 (43)	34 (52)
Somewhat satisfied	59 (24)	35 (23)	24 (26)	47 (29)	12 (15)	29 (28)	30 (22)	15 (21)	40 (26)	3 (19)	1 (33)	25 (30)	24 (26)	10 (15)
Neither satisfied nor dissatisfied	38 (7)	23 (15)	15 (16)	26 (16)	12 (15)	19 (18)	19 (14)	12 (17)	23 (15)	2 (12)	1 (33)	11 (13)	15 (16)	12 (18)
Somewhat dissatisfied	17 (7)	13 (9)	4 (4)	8 (5)	9 (11)	6 (6)	11 (8)	6 (8)	9 (6)	2 (12)	0 (0)	2 (2)	9 (10)	6 (9)
Very dissatisfied	11 (5)	7 (5)	4 (4)	7 (4)	4 (5)	5 (5)	6 (4)	7 (10)	4 (3)	0 (0)	0 (0)	1 (1)	6 (6)	4 (6)
Time to fall asleep														
15 min or less	137 (57)	83 (55)	54 (59)	89 (56)	48 (59)	58 (56)	79 (57)	32 (45)	94 (62)	9 (56)	2 (67)	50 (61)	50 (53)	37 (56)
16–30 min	72 (30)	48 (32)	25 (26)	46 (29)	26 (32)	29 (28)	43 (31)	25 (35)	39 (26)	7 (44)	1 (33)	24 (29)	30 (32)	18 (27)
31–60 min	26 (11)	15 (10)	11 (12)	19 (12)	7 (9)	12 (12)	14 (10)	9 (13)	17 (11)	0 (0)	0 (0)	8 (10)	11 (12)	7 (11)
> 60 min	7 (3)	4 (3)	3 (3)	6 (4)	1 (1)	4 (4)	3 (2)	5 (7)	2 (1)	0 (0)	0 (0)	0 (0)	3 (3)	4 (6)
Having trouble staying asleep														
None	86 (36)	49 (33)	37 (40)	61 (38)	25 (30)	44 (43)	42 (30)	28 (39)	54 (36)	3 (19)	1 (33)	**37 (45)**	**38 (40)**	**11 (17)**
1 or 2 times per week	108 (45)	69 (46)	39 (42)	77 (48)	31 (38)	44 (43)	64 (46)	28 (39)	70 (46)	10 (62)	0 (0)	36 (44)	40 (43)	32 (48)
3 or 4 times per week	21 (9)	16 (11)	5 (5)	10 (6)	11 (13)	6 (6)	15 (11)	5 (7)	14 (9)	1 (6)	1 (33)	6 (7)	8 (9)	7 (11)
5–7 days per week	27 (11)	16 (11)	11 (12)	**12 (8)**	**15 (18)**	9 (9)	18 (13)	10 (14)	14 (9)	2 (12)	1 (33)	**3 (4)**	**8 (9)**	**16 (24)**
Chronotype														
Evening type	93 (38)	53 (35)	40 (43)	**77 (48)**	**16 (20)**	**56 (54)**	**37 (27)**	**26 (37)**	**66 (43)**	**1 (6)**	**0 (0)**	**34 (41)**	**48 (51)**	**11 (17)**
Intermediate type	81 (33)	47 (31)	34 (37)	55 (34)	26 (32)	29 (28)	52 (37)	22 (31)	47 (31)	11 (69)	1 (33)	27 (33)	31 (33)	23 (35)
Morning type	68 (28)	50 (33)	18 (20)	28 (18)	40 (49)	**18 (17)**	**50 (36)**	23 (32)	39 (26)	4 (25)	2 (67)	**21 (26)**	**15 (16)**	**32 (48)**

*Note:* Bold values represent significant differences in the given category between groups and quartile calculated chronotype is presented.

Among all athletes, 90 (35%) were categorised in the category of no clinical sleep problems, 111 (43%) were in the mild clinical sleep problem category, 41 (16%) in the moderate clinical sleep problem category and 15 (6%) in the severe clinical sleep problems category (Table 3). No significant differences were found in sex, sports type, sports environment, competitive level, ASSQ chronotype, performance issues when travelling and sleep disordered breathing between different SDS categories (Table 3). There were significant differences in SDS category proportions between athletes who had sleep disturbances and athletes without sleep disturbances when travelling. Athletes with sleep disturbances when travelling had a higher proportion in the moderate and severe SDS categories (*p* < 0.001) (Table 4).

**TABLE 4:** SDS categories by sex, sports type, sports environment, competitive level, chronotype, sleep disturbances when travelling, performance, issues when travelling and sleep‐disordered breathing.

	None		Mild		Moderate		Severe		*p* (Cramer's V)
	*n*	%	*n*	%	*n*	%	*n*	%	
All athletes	90	35%	111	43%	41	16%	15	6%	
Sex									0.93 (0.04)
Male	54	36%	65	43%	24	16%	7	5%	
Female	31	34%	43	47%	13	14%	5	5%	
Sports type									0.52 (0.09)
Team Sport	58	36%	74	46%	21	13%	7	4%	
Individual Sport	27	33%	34	42%	16	20%	5	6%	
Sports Environment									0.07 (0.17)
Indoor Sport	36	35%	49	47%	10	10%	8	8%	
Outdoor Sport	49	35%	59	42%	27	19%	4	3%	
Competitive Level									0.36 (0.12)
Tier 2	19	27%	31	43%	17	24%	4	6%	
Tier 3	60	39%	67	44%	17	11%	8	5%	
Tier 4	5	31%	9	56%	2	12%	0	0%	
Tier 5	1	33%	1	33%	1	33%	0	0%	
Chronotype (ASSQ)									0.21 (0.13)
Morning‐type	9	29%	16	52%	5	16%	1	3%	
Intermediate‐type	43	36%	49	42%	23	19%	3	3%	
Evening‐type	33	29%	43	52%	9	16%	8	3%	
Sleep disturbance when travelling									**< 0.001 (0.32)**
Have sleep disturbance	11	22%	17	33%	17	33%	6	12%	
No sleep disturbance	74	39%	91	47%	20	11%	6	3%	
Performance issue when travelling									0.06 (0.17)
Have performance issue	6	21%	12	41%	8	28%	3	10%	
No performance issue	79	37%	96	45%	29	14%	9	4%	
Sleep‐disordered breathing									0.31 (0.12)
Have sleep‐disordered breathing	9	25%	16	44%	8	22%	3	8%	
No sleep‐disordered breathing	76	37%	92	45%	29	14%	9	4%	

*Note:* Quartile calculated chronotype is presented. Statistical differences were set at *p* < 0.05 (significant *p*‐value is highlighted with bold characters).

Abbreviation: ASSQ, Athlete Sleep Screening Questionnaire.

#### 3.3.2 Chronotype

Through the quartile analysis, 93 (38%) were classified as evening types, 81 (33%) as intermediate and 68 (28%) as morning types. Through the top (P90) and bottom (P10) percentile analysis to capture the extreme chronotypes, 28 (12%) were classified as evening types, 183 (75%) as intermediate and 31 (13%) as morning types. The r‐MEQ classified 35 athletes (14%) as evening types, 163 (67%) as intermediate and 44 (18%) as morning types. Cohen's kappa between quartiles ASSQ chronotype and r‐MEQ chronotype classifications was 0.43 (moderate). Cohen's kappa between top and bottom ASSQ chronotype classification and r‐MEQ chronotype classifications was 0.65 (good). To evaluate differences in sleep when travelling between chronotype extremes, we compared responses to ASSQ item 12 (sleep difficulties when travelling) for participants in the 10th and 90th percentiles of the chronotype distribution. Results indicated that a higher proportion of participants in the morning chronotype group reported experiencing sleep difficulties when travelling (*p* = 0.045).

The SDS scores by chronotype and the distribution of perceived sleep duration, sleep satisfaction, time to fall asleep and troubles staying asleep grouped by ASSQ chronotype is presented in Figure 1. No significant differences were found for SDS between chronotypes and rankings for sleep satisfaction, time to fall asleep and frequency of having trouble falling asleep. These results were confirmed when chronotype analysis was performed using r‐MEQ results.

**FIGURE 1:**
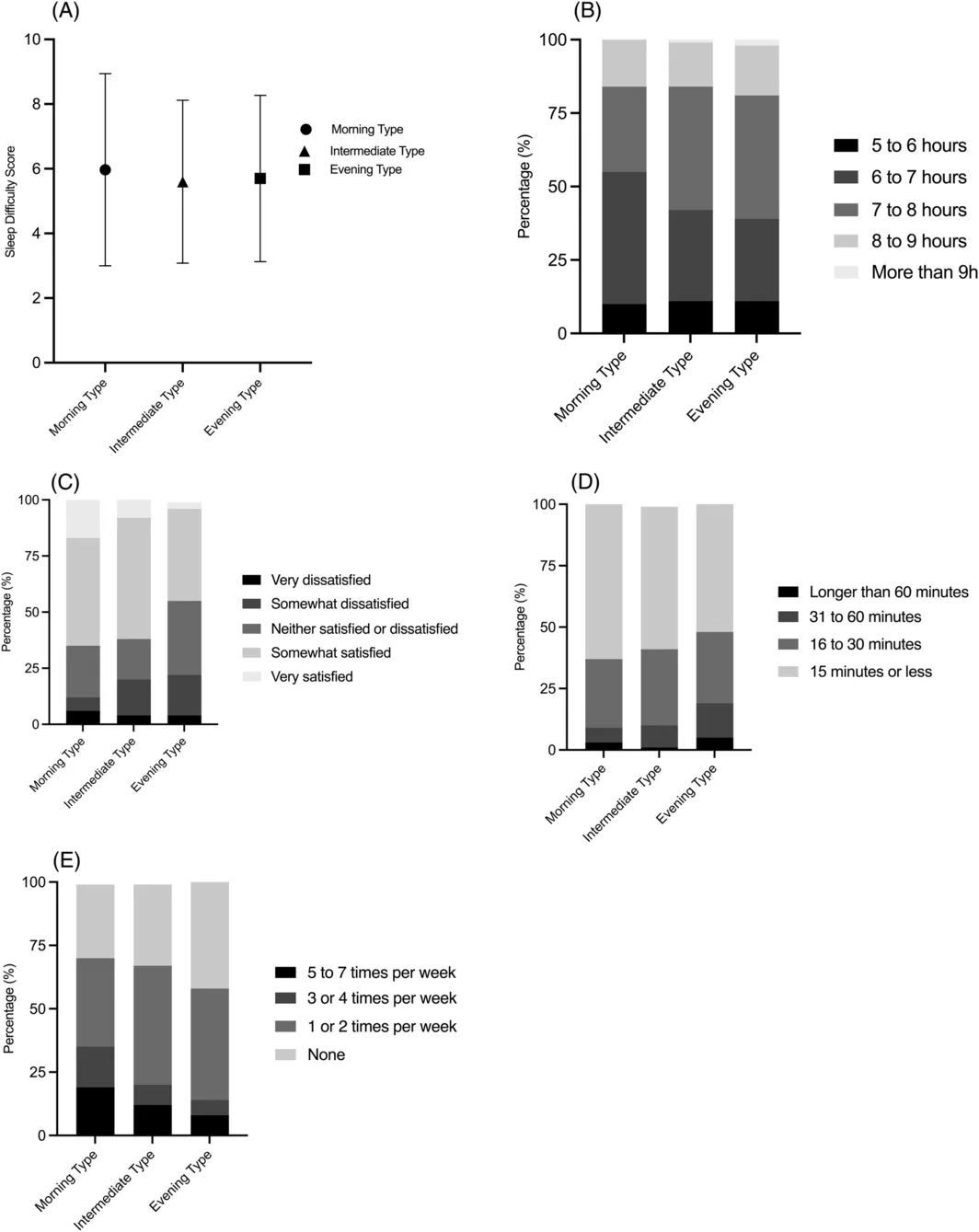
Participants' sleep characteristics by quartile chronotype: (A) sleep difficulty score, (B) nocturnal sleep duration, (C) sleep satisfaction, (D) time to fall asleep, and (E) trouble staying asleep.

## 4 Discussion

This study set out to both adapt the Athlete Sleep screening Questionnaire (ASSQ) to European Portuguese and explore the sleep health of Portuguese athletes through its application. More than 200 athletes from 22 sports completed a comprehensive questionnaire battery, enabling the validation of the Portuguese ASSQ and offering a detailed picture of sleep disturbances within this specific population.

In the first phase of the study, the translation process of the ASSQ was successfully completed, achieving a final Portuguese version of the ASSQ approved by all researchers involved. The internal consistency (Cronbach's alpha) for the SDS and chronotype was higher or similar to what was reported in the Arab language translation (SDS *α* = 0.43; chronotype subscale *α* = 0.63) (Alhowimel et al. 2023). On the other hand, the values were slightly lower than those reported in the Persian language translation (SDS *α* = 0.77; chronotype subscale *α* = 0.74) (Najafabadi et al. 2024). The fit indices from the CFA indicated a good fit. Altogether, the results of the EFA and CFA are in line with other studies that translated ASSQ to Persian (Najafabadi et al. 2024), Chinese (Zhang et al. 2024) and Arab (Alhowimel et al. 2023) languages. Thus, we considered that our results are acceptable and the validity of the Portuguese ASSQ was achieved.

Considering the whole sample, the average SDS was 5.7, which was similar to what was reported in a large sample of college athletes (5.8) (Rabin et al. 2020), slightly higher than Chinese athletes (5.3) (Zhang et al. 2022) and higher than a sample of Persian athletes (4.7) (Najafabadi et al. 2024). Comparable to the results of Zhang et al. (2022) in Chinese athletes, we did not find any significant differences in SDS between sex, sports type, sports environment and competitive level. However, we did find significant differences between age groups. Additionally, no differences were also found by SDS category, despite athletes of the highest tier reporting a much higher SDS than athletes from Tier 3 and 4; confidence intervals were much larger. While Hrozanova et al. (2021) previously reported that female endurance athletes tend to report worse subjective sleep quality compared to male athletes, that was not verified in our study, and the studies of Rabin et al. (2020) and Zhang et al. (2022). Furthermore, the fact that no differences were found between sports type or sports environment may also suggest that the ASSQ can be used to assess sleep disturbances of athletes in different sports with generalisation (Rabin et al. 2020).

While, to date, very few studies have utilised the ASSQ to describe sleep and assess sleep disturbances in large athletic cohorts, some do exist. In our study, 65% of athletes reported, at least, mild‐sleep problems and 22% reported moderate or severe sleep problems. Our results are comparable to those of Rabin et al. (2020) in American collegiate athlete and closely resemble the findings of Zhang et al. (2022) in Chinese athletes. However, in the latter study, the proportion of athletes with moderate or severe sleep problems was smaller (14%). Bender et al. (2018) reported that 25% of Canadian national team athletes had clinically significant sleep problems using the ASSQ. Sample differences may explain this (i.e., sports type, period of the season) (Halson 2014; Rabin et al. 2020). Also important to note when comparing these results, are possible differences due to participants' ethnicity (Zhang et al. 2022). While we did not account for that in our study (subjects were not inquired about ethnicity during the questionnaire battery), Huskey et al. (2021) applied the ASSQ in a sample of 1033 college athletes and found that black ethnicity athletes had worse sleep quality than the other two (white and other underrepresented ethnicities).

It is established that sleep disturbances may negatively affect physical performance, impairing reaction times, accuracy, sprint times and reducing endurance performance levels (Walsh et al. 2020; Gouttebarge et al. 2021; Halson 2014). In our study, athletes with sleep disturbances when travelling were more likely to report higher sleep problems (moderate or severe). While not significant, in athletes with performance issues when travelling, a higher proportion of moderate or severe sleep problems was also verified. Compared to the general population, athletes, especially high‐level and elite athletes, end up travelling more frequently (Walsh et al. 2020; Simpson et al. 2017). This can result in a higher risk of poor sleep health, with the appearance of nervousness and anxiety that result from travel (Simpson et al. 2017). Then, competition can cause insomnia and nightmares in athletes during sleep, which in turn may reduce sleep duration and sleep efficiency, ultimately affecting the overall sleep quality (Erlacher et al. 2011; Juliff et al. 2015).

Regarding the ASSQ chronotype subscale, our classification approach differed from the original ASSQ study and subsequent research using the same questionnaire. We opted to use a quartile or percentile (for extreme chronotypes) approach, aimed to capture the full range of chronotypes while accounting for their specific characteristics. In addressing athletes' sleep health, individual differences should be considered, as focusing solely on evening types for potential sleep disturbances overlooks the fact that morning types may also be susceptible to sleep problems. A major concern for elite athletes' sleep is jet lag, as they frequently travel across multiple time zones, making the acclimatisation process crucial for this population. Successful adjustment largely depends on the body's physiological response to light, which helps align circadian rhythms with the destination environment. Phillips et al. report substantial interindividual differences in melatonin suppression in response to light exposure (Phillips et al. 2019), which may influence how easily an athlete adapts to time zone changes. In our study, extreme morning types reported the greatest sleep difficulties when travelling, potentially reflecting these underlying differences in physiological light responses. As such, individual chronotype information is of great value when practitioners need to individualise strategies and adaptations for athletes. Additionally, when using questionnaires, it should also be noted that using pre‐established cutoffs may be a limitation when working with different populations, as so, average scores may be population/sample specific (Reis et al. 2020). Thus, a careful analysis of one's cohort should always be performed.

Regarding our chronotype results, using the quartile analysis we did not find any significant differences between morning, intermediate and evening types in the SDS categories and items relating to nocturnal sleep duration, sleep satisfaction, time to fall asleep, and trouble staying awake. In the SDS categories, the percentage of athletes with moderate or severe sleep problems was similar across chronotypes, although there was a higher number of evening type athletes experiencing severe problems. Nevertheless, it is important to note that society is largely structured around morning schedules, which makes evening types more vulnerable to circadian misalignment and sleep problems. Here, using the 0–4 cut‐off for extreme evening type, the same used in other studies, our results contrast with previous findings, where athletes identifying as evening types reported more frequent sleep disturbances (Zhang et al. 2022; Bender et al. 2019; Wang et al. 2024). Results from Chinese athletes showed that athletes with evening chronotype were more likely to be poorer sleepers (Zhang et al. 2022). Similarly, Bender et al. (2019) reported poorer sleep and sleep difficulties in evening chronotype athletes. In addition, Bender et al. (2019) found that misalignment of sleep times with circadian preference could contribute to poorer sleep quality in elite athletes. Previous studies have proposed chronotype to affect ratings of perceived exertion, fatigue scores and athletic performance as well as the quality of sleep after training and competition (Gupta et al. 2017; Vitale et al. 2017). However, it should be noted that the percentage of evening chronotypes using the ‘original’ cut‐off was much smaller in our study (12%), compared to Zhang et al. (2022) (26%), for instance. Nevertheless, considering past studies, coaches, support staff and physicians should pay attention to the athletes' chronotype and strategies should be individualised.

### 4.1 Limitations

Some limitations of the current study must be acknowledged. First, including a third phase of validity with a sleep physician and objective sleep assessment through polysomnography would strengthen the results of the study. Second, while the sample size included was relatively large, it can be considered small when compared to similar studies. However, it must be acknowledged that Portugal is a relatively smaller country compared to the United States or China, for example. Thus, the pool of athletes available to participate is also much lower. We did not collect other information that could also help further characterise athletes' sleep, such as training frequency and phase of the competitive season.

## 5 Conclusions

This study offers valuable insights for clinicians and practitioners to first identify maladaptive behaviours and chronotype among Portuguese athletes, and to further consider screening for possible sleep disturbances. The Portuguese version of the ASSQ demonstrated good psychometric properties, strong agreement with similar questionnaires, and ease of completion. We recommend using the chronotype dimension to characterise athletes, as shown by our findings that morning types experienced more sleep problems when travelling. A substantial proportion of athletes exhibited suboptimal sleep, with some likely to benefit from targeted interventions to improve sleep health. Further research should focus on validating the clinical relevance of the Portuguese ASSQ and extending its evaluation to a larger sample of high‐level athletes.

## Author Contributions

**João Barreira:** data collection, formal analysis, writing – original draft. **Júlio A. Costa:** conceptualisation, methodology, writing – review and editing. **João Brito:** supervision, conceptualisation, writing – review and editing. **Vasco Cardoso:** software, investigation, data collection, writing – review and editing. **Cátia Reis:** supervision, conceptualisation, methodology, writing – review and editing.

## Funding

We did not have specific research funding for this study however João Barreira was supported by the Portuguese Foundation for Science and Technology through a doctoral grant [2022.09446.BD].

## Ethics Statement

This study was approved by the Ethics Committee of the Portugal Football School (CEPFS 19.2023) and conducted in accordance with the ethical standards outlined in the Declaration of Helsinki.

## Conflicts of Interest

The authors declare no conflicts of interest.

## Supporting information


**Data S1:** Procedures of the translation, back‐translation and consolidation of the ASSQ.
**S2**. Athlete Sleep Screening Questionnaire description.
**S3**. Statistical analysis and psychometric validation of the Athlete Sleep Screening Questionnaire.
**S4**. Additional results of the validation analysis.

## Data Availability

The data that support the findings of this study are available on request from the corresponding author. The data are not publicly available due to privacy or ethical restrictions.
