# Supplementary material for: Sleep Health of Athletes of Different Sports: Translation and Validation of the Athlete Sleep Screening Questionnaire to Portuguese
Source: J Sleep Res. 2026 May 11;35(5):e70349. doi: 10.1111/jsr.70349 (PMC13619028; doi:10.1111/jsr.70349)
Supplement: Supplementary file 1 [file JSR-35-e70349-s001.docx]

**Sleep Health of Athletes of Different Sports: Translation and Validation of the Athlete Sleep Screening Questionnaire to Portuguese**

**Supplementary Material**

**S1. Procedures of the Translation, Back-translation, and Consolidation of the ASSQ**

We used the published item version of the ASSQ by Samuels and colleagues ^1^ after obtaining permission from the copyright owner of the questionnaire. The translation process comprised three sequential stages, following widely recommended guidelines for the cross-cultural adaptation of research instruments ^2,3^. These stages included an initial translation to European Portuguese, a back-translation of the Portuguese version into English, and a formal comparison of the back-translated versions with the original ASSQ. For the initial phase, two translators, bilingual native Portuguese speakers, fluent in English who work as professional translators, were asked to independently translate the ASSQ from English to Portuguese. The translated version was then reviewed by a panel of specialists in sleep research and sports medicine to assess content validity, ensuring conceptual clarity, cultural appropriateness, and relevance for the athletic population. Next, the Portuguese version was back-translated into English by two bilingual translators who are native English speakers and proficient in Portuguese. All translators involved were independent of the study authors, unfamiliar with the ASSQ, and unaware of the study’s objectives.

**S2. Athlete Sleep Screening Questionnaire Description**

The ASSQ is a 16-item questionnaire that was developed with the aim of examining 6 key sleep parameters: sleep quality, sleep duration, circadian preference, insomnia, sleep disturbances while traveling, and sleep-disordered breathing (i.e., obstructive sleep apnea) ^1^. A sleep difficulty score (SDS) was also developed within the questionnaire, based on the response to items 1, 3, 4, 5, and 6 with a higher score indicating higher sleep difficulty (and poorer sleep). Item 1: *During the recent past, how many of actual sleep did you get at night? (This may be different than the number of hours you spent in bed.)*; Item 3: *How satisfied/dissatisfied are you with the quality of your sleep?* Item 4: *During the recent past, how long has it usually takes you to fall asleep each night?* Item 5: *how often do you have trouble staying asleep?* Item 6: *During the recent past, how often have you taken medicine to help you sleep (prescribed or over-the-counter)?* Items 1 and 3 are scored from 0 to 4 and items 4, 5, and 6 are scored from 0 to 3. By adding the score of the 6 items, the SDS is then calculated, ranging from 0 to 17 points. This is used to classify participants into 4 different sleep difficulty categories: none (SDS 0-4), mild (SDS 5-7), moderate (SDS 8-10), and severe (SDS 11-17). Scores over 8 are representative of athletes who could benefit from further assessment and potentially sleep interventions.

Subject’s chronotype is also calculated, as a subscale of the ASSQ, based on the responses to item 7, 8, 9, and 10. Item 7: *Considering only your own “feeling best” rhythm, at what time would you get up if you were entirely free to plan your day?* Item 8: *How alert do you feel during the first half-hour after having awakened?* Item 9: *Do you consider yourself to be a morning type person or an evening type person?* Item 10: *Considering your own “feeling best” rhythm, at what time would you go to bed if you were entirely free to plan your evening?* Items 7 and 10 are scored from 0 to 4 and items 8 and 9 are scored from 0 to 3. The scores from the 4 items are added to create a final score that goes from 0 to 14.

**S3. Statistical Analysis and Psychometric Validation of the Athlete Sleep Screening Questionnaire**

Internal consistency was assessed using Cronbach’s α and McDonald’s Ω. A threshold of α > 0.70 was considered acceptable, while item-total correlations below 0.30 were reviewed for potential removal^4^.

Exploratory Factorial Analysis (EFA) was conducted using Principal Axis Factoring with oblimin rotation, as correlations between factors were expected. The Kaiser-Meyer-Olkin (KMO) measure of sampling adequacy and Bartlett’s test of sphericity were examined to determine factorability. The number of factors was determined based on the scree plot and parallel analysis. Then, based on EFA results and scree plot analysis, Confirmatory Factorial Analysis (CFA) were performed. Model estimation was conducted using the Maximum Likelihood Estimation method. Covariances between latent variables were estimated. Model fit was assessed using multiple indices: Chi-square test, Comparative Fit Index (CFI) and Tucker-Lewis Index (TLI) (Acceptable fit was defined as CFI & TLI > 0.90), Root Mean Square Error of Approximation (RMSEA) (Values below 0.08 indicated acceptable fit), and Standardized Root Mean Square Residual (SRMR) (values below 0.08 indicated good fit) ^5^. The final models were interpreted based on factor loadings, fit indices, and theoretical coherence.

**S4. Additional Results of the Validation Analysis**

*ASSQ Sleep Difficulty Score*

The KMO measure of sampling adequacy was 0.66, indicating that the sample is adequate for factor analysis. Bartlett's test of sphericity was significant (χ² = 143.0, p < 0.001), suggesting that the correlation matrix is not an identity matrix. A parallel analysis and scree plot indicated that two factors should be retained. The EFA was performed using principal axis factoring with varimax rotation. The two-factor solution accounted for 45% of the total variance, with MR1 explaining 23% and MR2 22%. Question 6 was dropped from the final model, having a factor loading below the 0.4 threshold. The two-factor model of the SDS scale obtained from the EFA was also validated based on the CFA. The CFA results indicate that the model has an excellent fit. Both factors are well-defined by their respective items, and the fit indices are all within acceptable ranges: χ²(2) = 2.97, p = 0.563, DF = 6, CFI = 1.0, TLI = 1.057, RMSEA = 0.0 (90% CI 0.0, 0.169), SRMR= 0.017.

*ASSQ Chronotype*

The KMO measure of sampling adequacy was 0.66, indicating that the sample is adequate for factor analysis. Bartlett's test of sphericity was significant (χ² = 123.5 p < 0.001), suggesting that the correlation matrix is not an identity matrix. A parallel analysis and scree plot indicated that one factor should be retained. However, after running CFA analyses aiming to improve the model, a 2-factor analysis was chosen. The EFA was performed using principal axis factoring with varimax rotation. The CFA results further support the proposed two-factor model of the chronotype questions. The fit indices indicate a good fit, although the RMSEA is slightly higher than the preferred threshold. The chi-square test was not significant, suggesting that the model is an adequate representation of the data. The standardized factor loadings for Factor 1 ranged from 0.560 to 0.862, indicating that the items Q7 and Q10 are strong indicators of this factor. For Factor 2, the factor loadings ranged from 0.487 to 0.713, showing that the items Q8 and Q9 are moderate to strong indicators of this factor. The significant covariance between Factor1 and Factor2 (ϕ = 0.694, p < 0.001) suggests that these factors are related, reflecting an underlying commonality in the chronotype questions but still capturing distinct aspects of the construct.

χ²(1) = 3.331, p = 0.068, DF = 6, CFI = 0.98, TLI = 0.883, RMSEA = 0.0 (90% CI 0.0, 0.250), SRMR= 0.027

Analyzing the PSQI seven components indicated (Cronbach’s alpha = 0.63 [95% CI 0.49, 0.67]) and r-MEQ (Cronbach’s alpha = 0.66 [0.58, 0.74] were below the defined suitable criterion (>0.7). A strong, positive correlation was found between SDS and PSQI scores (r=0.61 [95% CI 0.51, 0.67]). A strong, positive correlation was found between SDS chronotype subscale and the r-MEQ scores (r=0.85 [0.81, 0.88]).

*Sleep Difficulty Score and Chronotype: Ceiling and floor effects*

The Ceiling and Floor Effects for the SDS and chronotype were 8% and 1%, respectively, so the ceiling and floor effect was not observed. However, the final total scores for the SDS were not normally distributed.

**References**

1.	Samuels, C., James, L., Lawson, D. & Meeuwisse, W. The Athlete Sleep Screening Questionnaire: a new tool for assessing and managing sleep in elite athletes. *Br J Sports Med* **50**, 418–422 (2016).

2.	Beaton, D. E., Bombardier, C., Guillemin, F. & Ferraz, M. B. Guidelines for the process of cross-cultural adaptation of self-report measures. *Spine (Phila Pa 1976)* **25**, 3186–3191 (2000).

3.	Sperber, A. D. Translation and validation of study instruments for cross-cultural research. *Gastroenterology* **126**, S124-128 (2004).

4.	Tavakol, M. & Dennick, R. Making sense of Cronbach’s alpha. *Int J Med Educ* **2**, 53–55 (2011).

5.	Hu, L. & and Bentler, P. M. Cutoff criteria for fit indexes in covariance structure analysis: Conventional criteria versus new alternatives. *Structural Equation Modeling: A Multidisciplinary Journal* **6**, 1–55 (1999).
